# Radio-Frequency and Microwave Techniques for Non-Invasive Measurement of Blood Glucose Levels

**DOI:** 10.3390/diagnostics9010006

**Published:** 2019-01-08

**Authors:** Tuba Yilmaz, Robert Foster, Yang Hao

**Affiliations:** 1 Department of Electronics and Communication Engineering, Istanbul Technical University, 34469 Istanbul, Turkey; tuba.yilmaz@itu.edu.tr; 2 Department of Electronic, Electrical and Systems Engineering, University of Birmingham, Birmingham B15 2TT, UK; r.n.foster@ieee.org; 3 School of Electronic Engineering and Computer Science, Queen Mary University of London, London E1 4NS, UK

**Keywords:** blood glucose levels, non-invasive measurement, glucose-dependent dielectric properties, RF sensing, microwave resonators, microwave spectroscopy, dielectric spectroscopy, on-body antennas

## Abstract

This paper reviews non-invasive blood glucose measurements via dielectric spectroscopy at microwave frequencies presented in the literature. The intent is to clarify the key challenges that must be overcome if this approach is to work, to suggest some possible ways towards addressing these challenges and to contribute towards prevention of unnecessary ‘reinvention of the wheel’.

## 1. Introduction

The prevalence of Type 2 diabetes has been rapidly increasing through the latter half of the twentieth and into the twenty-first century. It has been associated with changes in life style during that period, including increasing adoption of unhealthy dietary habits and limited daily activity. In 2014, the prevalence of diabetes among adults older than 18 years globally had increased to 8.5%, from 4.7% in 1980 [1]. Mortality resulting directly from diabetes was estimated to be 1.6 million in 2015. Diabetes is a chronic condition and must be managed well to prevent complications of the disease, including (but not limited to) cardiovascular disease, blindness, kidney failure, increased risk of stroke and lower limb amputation [2]. A large number of techniques have been considered for non-invasive glucose monitoring, including the analysis of sweat, urine (e.g., [3]), tears (e.g., [4]), and saliva (see the recent review in [2]), breath analysis (relating blood glucose levels to acetone, which is produced during ketosis [5,6,7,8]), as well as various spectroscopic methods, with limited or no success to date [2,9,10]. Furthermore, few of these techniques are suitable for continuous monitoring (particularly those requiring samples of saliva, urine and breath), although potentially still of use for non-invasive validation, or even calibration, of other wearable sensors.

Currently, blood glucose levels are mostly monitored with ambulatory monitoring devices, where a drop of blood has to be drawn via a lancet and placed onto a chemically pre-treated strip inserted in a device [2]. When the blood is dropped onto the strip, the glucose creates a low-level current. The monitoring device quantifies the blood glucose level via the intensity of the current. These ambulatory devices suffer from error rates as high as 20% for older devices and 15% for devices meeting the current International Standards Organisation standard for blood-glucose monitoring systems [10,11]. Furthermore, these devices measure the capillary blood glucose levels (since the blood sample is usually drawn from finger tips, or sub-cutaneous measurements using ‘needle-patches’ placed on the torso for more modern ‘continuous monitoring’ systems) and it is known that the capillary blood glucose levels lags behind the actual glucose levels (e.g., [2,10]). The main disadvantage of the current ambulatory monitoring devices are the invasiveness, after the relatively recent development of commercial ‘continuous monitoring’ systems (such as Dexcom’s G6 system, Abbott’s FreeStyle Flash/Libre system and Medtronic’s Guardian systems, some of which have sensor lifetimes up to 14 days [10]). Off-the-shelf monitoring devices have a number of practical obstacles, however, for example, the blood glucose levels is known to be affected from the sanitation of the measurement site, it is advised that the measurement site should be washed with warm water to increase the circulation, the patient is vulnerable to infections, the tissue becomes increasingly deformed at the measurement site over time and the cost of the measurement. Therefore, there is a need for new technologies that can offer reliable and continuous measurements while being unobtrusive to the patient and reducing costs. It is worth noting that blood glucose monitoring would also be of interest for non-diabetic people, such as astronauts, elite athletes and security personnel.

One such technique that has captured the attention of researchers is the RF/microwave sensing of blood glucose levels. Within the last decade, with the development of wireless technologies, an increased interest in the interaction of electromagnetic waves and biological tissues has emerged. With the motivation of designing medical diagnostic and therapeutic devices, many studies focussed on characterization of dielectric property profiles of biological tissues and anomalies, with applications including burn or wound monitoring and detection of cancerous tissue (for example, [12,13,14]). The possibility of using radio-frequency (RF) or microwave sensing for blood glucose level characterization has also been investigated during this period (in fact, such technologies have been investigated throughout the twenty-first century at least, including various unsuccessful attempts at commercialisation; an interesting perspective on research into non-invasive glucose monitoring can be found in [10]). In this paper, we review the studies conducted to investigate the interaction of electromagnetic waves with glucose molecules, covering frequencies between approximately 1 kHz and 100 GHz, with a focus on ‘microwave’ frequencies (which we take to mean 0.1–20 GHz in this paper). We will mostly use the term ‘microwave spectroscopy’ to describe this technique, but will also use dielectric spectroscopy interchangeably (although the latter is the more general). A related term, impedance spectroscopy, is used at low radio frequencies (below about 1 MHz), where it is more convenient to represent the material properties as resistances, capacitances and inductances. As these are related directly to the complex permittivity (and complex permeability, where relevant), we will mostly avoid this terminology to avoid confusion.

The paper is organized as follows: we begin by examining the glucose-dependent properties of human tissues, with the intent being to highlight the first major obstacle to non-invasive monitoring via microwave spectroscopy, sensitivity. We also briefly discuss the second major issue, selectivity, or how to attribute measured changes in dielectric properties to changes in blood glucose. We then review the various frequencies used in the literature and discuss how the operating frequency might be chosen in the light of the sensitivity and selectivity issues. A survey of resonators and antennas used in the literature follows, with some comments based on the frequency and mode of operation and the sensitivity and selectivity issues. We conclude with a brief discussion of possible ways to meet the sensitivity and selectivity challenges.

## 2. Glucose Dependent Dielectric Properties

The dielectric properties of a material govern the wave behaviour in that medium. Therefore, the dielectric properties of a medium are one of the primary design parameters of RF/microwave structures. The growth of, first, mobile (cellular) communications, followed by body-centric communications (including various wearable and implantable communications devices), together with the possibility of using such devices for physiological monitoring, has encouraged a great deal of interest in how the human body affects electromagnetic waves, whether for health and safe exposure, maintaining links between a cellular phone and base station or maintaining links between that same phone and a Bluetooth headset [15]. To provide the necessary database, the dielectric properties of biological tissues, including those with biological anomalies, have been extensively reported in the literature [16,17,18,19]. Since the success of microwave diagnostics and treatment applications depend on the dielectric property discrepancy between the normal and abnormal tissues (e.g., [13,14]), the evaluation of the dielectric properties is critical. Recently, the possible application of microwaves for non-invasive and continuous blood glucose monitoring has motivated many researchers to investigate the glucose-dependent dielectric properties of blood and other liquids. To this end, dielectric properties of blood plasma, blood, saline solutions and deionized water have been reported in the literature. The remainder of this section summarizes the reported literature on glucose-dependent dielectric properties. We first provide a brief primer on terminology.

The permittivity is a complex quantity, with the imaginary part representing the loss, which includes heating and conductive effects. Key terms include:

- permittivity—a bulk (that is, volume-average) material property quantifying the ability of the medium to store electrical energy;
- electric constant—also called the vacuum permittivity or permittivity of free space, this is the permittivity for an ideal vacuum and a physical constant;
- relative permittivity—the permittivity of a medium normalised by the electric constant (this is often applied to the real part of the normalised permittivity only);
- dielectric loss factor—another name for the imaginary part of the permittivity (often referring to the imaginary part of the normalised permittivity);
- effective permittivity—the permittivity of a composite (heterogeneous) material (for example, a layered structure, where each homogeneous layer has different properties) represented as an equivalent homogeneous medium (this could be used for the relative effective permittivity, which should be evident from context);
- loss tangent—a means of representing the loss in a dielectric as the ratio of imaginary to real parts (usually denoted ‘tan(δ)’);
- conductivity—the ability to transfer charge, which is a loss mechanism for dielectrics;
- phantom—a digital or physical object that allows the parameter of interest to be changed in a controlled manner;
- tissue-mimicking material—a material designed to have the same dielectric properties as the tissue of interest, for use in physical phantoms;
- Q factor—a term used to quantify the performance of resonators, where greater Q-factors imply stronger resonances and more narrow bandwidths. A distinction is made between the ideal (‘unloaded’) performance and the ‘measured’ (‘loaded’) performance;
- resonant frequency—strictly, this is the frequency at which the input impedance of a resonator is purely real (resistive); in practice, this can be used for the frequency of a maximum (transmit-mode) or minimum (reflect-mode) of the resonator response. Changes in the dielectric properties ‘loading’ the resonator can affect some or all the resonant frequency, the bandwidth at resonance and the magnitude of the resonance (maximum or minimum) in detectable amounts.

### 2.1. Measurements Performed with Biological Tissues

In [20], glucose levels of blood plasma, collected from ten adults with ages ranging from 18 to 40 years, were changed in-vitro by adding 5% dextrose solution to the plasma to achieve values between 0 mg/dL to 16,000 mg/dL. The blood glucose concentration was changed by doubling the previous level; that is, first the plasma glucose levels were increased from 0 mg/dL to 250 mg/dL, then from 250 mg/dL to 500 mg/dL, etc. The dielectric properties of the blood plasmas were measured for each glucose level between 0.5 GHz and 20 GHz. A meaningful change was observed when the glucose levels were increased from 2000 mg/dL to 4000 mg/dL. The dielectric properties were measured with Agilent’s Open-ended Coaxial Dielectric Probe Kit [21]. As a continuation of this study, the Cole–Cole equation was fit to the collected dielectric property data [22]. The Cole–Cole equation is a mathematical expression that has been utilized in the literature to model dielectric property behaviour with a minimum number of variables. In [18], the four-pole Cole–Cole equation was utilized to model the dielectric properties of biological tissues; however, it was concluded in more recent studies that a single-pole Cole–Cole equation is adequate to model dielectric property behaviour of biological tissues over an ultra-wide frequency range. The single-pole equation is [23]:

(1) $$\hat{\varepsilon }\left(\omega \right)=\varepsilon_{\infty }+\frac{\varepsilon_{s}-\varepsilon_{\infty }}{1+{\left(j\omega \tau \right)}^{\left(1-\alpha \right)}}+\frac{\sigma_{i}}{j\omega \varepsilon_{0}}$$

where $\varepsilon_{\infty }$ is the relative permittivity at field frequencies, $\varepsilon_{s}$ is the static permittivity, τ is the relaxation time for a dispersion region, α represents the broad distribution of the relaxation time constant and $\sigma_{i}$ is the ionic conductivity. The difference $\varepsilon_{s}-\varepsilon_{\infty }$ is denoted as Δε and the effective permittivity $\hat{\varepsilon }$ is a function of frequency f=ω/2π.

In [22], after fitting the Cole–Cole parameters to blood plasmas with different glucose concentrations, the Cole–Cole parameters were represented as polynomial equations to depict the glucose-dependent change of dielectric properties. These polynomial equations for the Cole–Cole parameters are used in this work with the intention to demonstrate quantitatively the dielectric property behaviour of tissues when changing the glucose level. The polynomial equation is a quadratic, of the form:

(2) $$u\left(\chi \right)=a_{n}\chi^{2}+b_{n}\chi +c_{n}$$

with *u* a dummy variable representing a given parameter and χ representing the glucose concentration. Coefficients $a_{n}$, $b_{n}$ and $c_{n}$ are given in [22] for each Cole–Cole model parameter. The polynomials with numerical coefficients are also given below:

(3) $$\varepsilon_{\infty }\left(\chi \right)=0.99\times 10^{-2}\times \chi^{2}+0.47\times 10^{-1}\times \chi +2.3$$

(4) $$\Delta \varepsilon \left(\chi \right)=0.93\times 10^{-2}\times \chi^{2}-0.21\times \chi +71.0$$

(5) $$\tau \left(\chi \right)=0.12\times 10^{-2}\times \chi^{2}+0.23\times \chi +8.7$$

(6) $$\sigma_{i}\left(\chi \right)=0.63*10^{-2}*\chi^{2}-0.14*\chi +2.0$$

**Figure 1:**
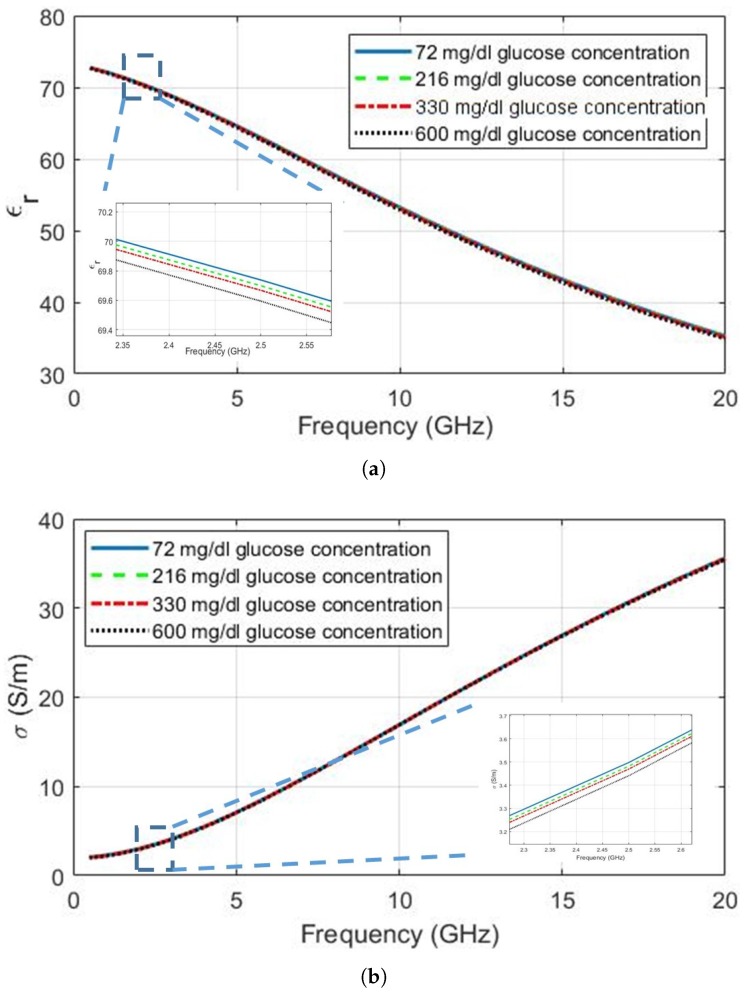
Dielectric properties of blood plasma with glucose variations graphed with Cole–Cole parameters polynomials given in [22]: (**a**) relative permittivity $\epsilon_{r}$; (**b**) conductivity σ.

The Cole–Cole equations are plotted using the polynomials and coefficients for glucose concentrations of 72 mg/dL, 216 mg/dL, 330 mg/dL and 600 mg/dL (equivalently, 4 mmol/L, 12 mmol/L, 18.3 mmol/L and 33.3 mmol/L, respectively). Figure 1a,b show the change in relative permittivity and conductivity, respectively. This range of glucose concentrations was chosen based on the following: first, the blood glucose of a healthy human changes between 72 mg/dL to 216 mg/dL; second, a glucose level of 330 mg/dL was reported in [24] for a diabetic patient and is here used as a realistic value that must be detectable; and the maximum glucose level that can be measured by the current commercial ambulatory monitoring devices [25], 600 mg/dL. It can be seen that both relative permittivity and conductivity decrease as the glucose concentration increases from 72 mg/dL to 600 mg/dL. More importantly, however, it is evident that the sensitivity of the dielectric properties to realistic values is extremely small, being around 0.2 units for the relative permittivity and 0.1 S/m for the conductivity. The decrease in relative permittivity with the increase in blood glucose levels is slightly greater than the decrease in conductivity, but monitoring of both parameters may still be required. Furthermore, highly sensitive sensors will be required to detect small changes if continuous monitoring is desired, especially when including the effects of system noise and other factors that also affect the tissue dielectric properties. Although [22] presents a model to represent the glucose-dependent dielectric properties, the Cole–Cole parameter fittings show that $\varepsilon_{\epsilon }$ increases with the increase in glucose levels. This is not consistent with the measurement results and the fitting provided for the Δϵ. Nevertheless, to the best of the authors’ knowledge, both the approach and utilization of blood plasma provides the most realistic insight to the glucose-dependent dielectric property change.

In another study [24], blood samples were collected from twenty patients (two per patient), where eight of the patients were diabetic and twelve of the patients were non-diabetic. One blood sample per volunteer was placed in a vial containing Ethylenediaminetetraacetic acid, utilized to prevent blood clots forming in the withdrawn sample; the other samples were kept without additives. The volume of each blood sample was 3 mL. The blood samples were then transferred to 5-mL dishes and the dielectric properties of the samples in the dish containers measured with Agilent’s high temperature dielectric probe kit [21]. The blood glucose levels of the collected blood samples varied between 79 mg/dL to 330 mg/dL. A modified two pole Cole–Cole model was fit to the collected dielectric property measurements. It was concluded that the relative permittivity of blood drops five units between 80 mg/dL and 140 mg/dL. It should be noted that the measurement results were noisy and this could be due to the small sample size. The sensing volume of the probe could be larger than the sample thickness. Also, the number of measurements was not given in detail in the reported study.

### 2.2. Measurements Performed with Phantom Materials

Performing measurements using biological tissues is necessary for obtaining realistic results, but carries legal, ethical and financial implications. In addition, the complexity of real tissues can make the task of interpreting measurements challenging. The use of phantoms in place of tissues is common, particularly in the earlier stages of research and product development, to minimise these issue, although in vivo studies will always be required at some stage. In one study [26], tissue-mimicking phantoms replicating the dielectric properties of blood and other tissues were fabricated with oil-in-gelatin dispersion phantoms to quantify the realistic glucose-dependent dielectric properties. Oil-in-gelatine dispersion phantoms are used in the literature to imitate the dielectric properties of all biological tissues [27]. Such phantoms mainly include deionized water, gelatin, oil, a surfactant (e.g., dishwasher detergent) and salt (NaCl). The gelatin helps to solidify the phantom, deionized water is both used for dissolving the gelatine and increasing the dielectric properties. The surfactant is used increase the homogeneity when mixing the oil and other ingredients. Oil has very low dielectric properties and thus, decreases the dielectric properties of the mixture; finally, NaCl is utilized for increasing the conductivity of the phantom. Most (all) human body tissues can be obtained by using the same base recipe; however, the amounts of the ingredients have to be adjusted to obtain the desired tissue dielectric properties. For high water-content tissues, the amount of oil should be reduced and the reverse is true for low water-content tissues. Examples of the recipes for fat-, skin-, muscle- and blood-mimicking materials are given in Table 1 [26].

After characterizing the blood-mimicking material, different amounts of powdered dextrose was added to four blood-mimicking phantom materials. Note that the dextrose is the naturally available form of glucose in the blood and it can either be obtained in powder form or dissolved in water. The amount of dextrose added to the phantoms is equivalent to the blood glucose levels between 0 mg/dL and 216 mg/dL. This captured the realistic dielectric property change, since the blood glucose levels of a healthy person varies between 72 mg/dL and 216 mg/dL. The phantoms were prepared and left overnight to solidify. Next, the dielectric properties of the phantoms were measured with Agilent’s high temperature open-ended coaxial dielectric probe kit. The measurements were repeated twenty times for each phantom and the median values of the measurements were taken as the dielectric properties of the phantom materials. This process was important, since the commercially available open-ended coaxial probes suffer from high measurement error rates, 5% for off-the-shelf open-ended coaxial probes [21]. According to the authors’ experience, the reported error rate may increase depending on the equipment wear off, cable type, calibration quality and cable/probe movements. Considering that the change of blood glucose levels does not result in high dielectric contrast, the accuracy of the dielectric property measurements becomes critical to quantify the glucose-dependent dielectric property change.

A one-pole Cole–Cole equation, given by (1), was fit to the measured dielectric properties to quantify the glucose-dependent dielectric property change [26]. The accuracy of the fitting was checked by calculating the Euclidean distance, given in Equation (7). The accuracy of the fitting is also critical to minimize error that would hinder accurate detection of the dielectric property change due to a change in glucose levels.

(7) $$e=\frac{1}{N}\sum_{i=1}^{N}\left[{\left(\frac{\varepsilon_{\omega_{i}}^{\prime }-{\hat{\varepsilon }}_{\omega_{i}}^{\prime }}{median\left[\varepsilon_{\bar{\omega_{i}}}^{\prime }\right]}\right)}^{2}+{\left(\frac{\varepsilon_{\omega_{i}}^{\prime \prime }-{\hat{\varepsilon }}_{\omega_{i}}^{\prime \prime }}{median\left[\varepsilon_{\bar{\omega_{i}}}^{\prime \prime }\right]}\right)}^{2}\right]$$

where $\varepsilon_{\omega_{i}}^{\prime }$ and $\varepsilon_{\omega_{i}}^{\prime \prime }$ are the measured real and imaginary parts of the permittivity, ${\hat{\varepsilon }}_{\omega_{i}}^{\prime }$ and ${\hat{\varepsilon }}_{\omega_{i}}^{\prime \prime }$ are the equivalent fitted dielectric properties and *N* is the number of points used within the measurement frequency range.

**Figure 2:**
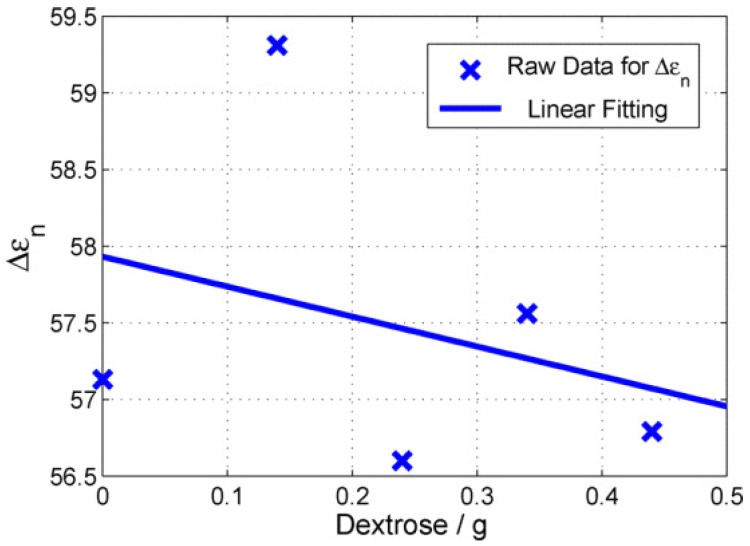
Glucose-dependent change in Δϵ parameter collected from the blood-mimicking phantoms with glucose levels ranging from 0 mg/dL to 216 mg/dL, reported in [26].

When the fitted Cole–Cole parameters were analysed, it was seen that the Δϵ parameter decreased with the increase in the dextrose levels in the blood-mimicking phantoms. This is consistent with the previously reported results, where it was concluded that the increase in dextrose levels decreases the permittivity of the blood plasma [20]. The change in Δϵ parameter is shown in Figure 2; it can be seen that Δϵ parameter changed by one unit when the dextrose levels increased from 0 mg/dL to 216 mg/dL. This again emphasises how the sensitivity of the measurement system will be critical in successfully tracking changes in blood glucose level, particularly in continuous measurement scenarios.

The authors of [28] also used recipes for blood mimicking phantom materials (proposed in [29]). The phantoms were composed with water, salt, flour and sugar [28]. The recipe for the blood-mimicking material required 66.9%, 0.8%, 25.0%, and 7.3% by weight for water, salt, flour, and sugar, respectively [28]. A change of glucose level was emulated by adding varying amounts of sugar. In particular, the results from real blood samples (Section 2.1) were used to predict the equivalent amount of sugar to use in the phantom recipe (in place of the amount specified in the provided phantom recipe [28]). Reasonable agreement at the higher frequencies used (approximately 2.2–6 GHz) were observed between the blood measurements and phantoms fabricated for all sugar amounts (two phantoms were made for each amount of sugar), with greater divergence at the lower frequencies.

Most of the reported work in the literature was performed to characterize the glucose-dependent dielectric property change with broadband measurement techniques; namely, open-ended coaxial probes. Although the technique offers a number of advantages, including minimal sample preparation requirements and broadband measurement capabilities, it suffers from greater measurement errors. Therefore, a more narrow-band resonator technique was employed in [30] to retrieve the dielectric properties of phantom materials made using flour, water and sugar (note that these phantoms were not blood mimicking material, but were used to represent the lossy medium of the human body). With the proposed analytical method, highly accurate permittivity results were obtained, despite the fact that the resonator was not fully optimised for this task. The loss in the phantom (human body) acts to increase the bandwidth of the resonator, increasing the noise included with the measurement and reducing sensitivity. An extremely narrow-band (high Q-factor) resonator may give even greater sensitivity, also improving accuracy. Importantly, the methodology employed [30] gave accurate results despite the limitations of the narrow-band resonator employed.

### 2.3. Measurements Performed with De-Ionized Water

In an attempt to simplify the sample preparation, some research groups approximate blood with water. In some studies, this was justified by the fact that the blood plasma is a high water-content tissue and, since the mineral percentages in blood plasma are very low, the minerals can be ignored. Others argued that a ‘physiological solution’ (0.9% NaCl solution) tends to imitate blood and can thus, be used as a base for measuring the change in dielectric properties with respect to change in glucose levels. The rest simply investigated the glucose-dependent dielectric properties of de-ionized water, since it is free of other interactions and this helps with quantifying the effect of only glucose to dielectric properties.

In [31], blood-simulating solutions were obtained by adding 10, 20, 30, 40 and 50 percent-by-weight table sugar into the de-ionized water. The dielectric properties of the solutions were measured from 200 MHz to 5 GHz. As expected, the permittivity of the solutions decreased with the increase in sugar levels, whereas the conductivity increased. Essentially, this work shows the macro trends in dielectric property behaviour with respect to changes in sugar levels. However, this may not represent the dielectric property behaviour for realistic glucose levels.

In [32], the dielectric properties of physiological solutions and de-ionized water were measured, having seven different glucose amounts varying from 0 mol/L to 6 mol/L at 1 mol/L increments (that is, from 0 mg/dL to 108,000 mg/dL with 18,000 mg/dL increments). As noted earlier, the normal range of glucose is between 72 mg/dL and 216 mg/dL for a healthy person. A Type 2 diabetes patient can experience higher blood glucose levels. Ambulatory devices are unable to measure glucose levels above 600 mg/dL [25]. If the blood glucose levels are above 600 mg/dL, the patient is at high risk of experiencing hyperosmolar hyperglycaemic state (HHS). Therefore, an increase of 18,000 mg/dL is much higher than the realistic glucose level changes. Also, this study does not specify the type of glucose used to obtain the mixtures. The reported measurement results confirm that the increase in the glucose levels decreases the relative permittivity of the mixture at lower frequencies. However, it concludes that, at higher frequencies, the increase in glucose levels increases the relative permittivity of the mixture. For conductivity, it was concluded that the increase in glucose levels has an effect on conductivity and this effect is frequency-dependent; also, the amount of change in conductivity is smaller when compared to relative permittivity. It should also be noted that the reported measurements were performed with Agilent’s open-ended coaxial performance probe between 500 MHz and 67 GHz. The technical specifications indicate that the probe is reliably operational between 500 MHz and 50 GHz [21]. Therefore, the reported measurements above 50 GHz are higher than the recommended operation range of the probe. Unlike other reported studies, the measurements presented in [32] were performed at 37 ${}^{{}^{\circ}}$C (that is, at human body temperature). It was concluded that the sensitivity of both relative permittivity and conductivity values to the glucose was between 0.01% to 0.02% per mmol glucose change in one litre of blood-mimicking material. Considering that the relative permittivity of blood in the 2.45 GHz license-exempt Industrial, Scientific and Medical (ISM) band is 58.2 units [17,18], the relative permittivity change will be 0.05 units if the blood glucose is increased from 72 mg/dL to 216 mg/dL.

The dielectric properties of glucose–water solutions were used to model the glucosedependent dielectric property change at 25 ${}^{{}^{\circ}}$C in [33,34,35], with glucose concentrations between 0 mg/dL and 16,000 mg/dL. The measurements were performed with an in-house fabricated open-ended coaxial probe and an in-house algorithm. The dielectric properties were retrieved using artificial neural networks that fit the Debye model to the measurements for each glucose concentration. The Debye model is very similar to the Cole–Cole model; in the Debye model, the α parameter in Equation (1) is zero and the ionic conductivity is also ignored ($\sigma_{i}=0$). The Debye parameters were then again expressed as polynomials with variables representing the change in glucose levels, with the same basic polynomial model given in Equation (2) used. The coefficients of the polynomials are given in Table 2 for $\varepsilon_{\infty }$, $\varepsilon_{s}$ and τ.

**Figure 3:**
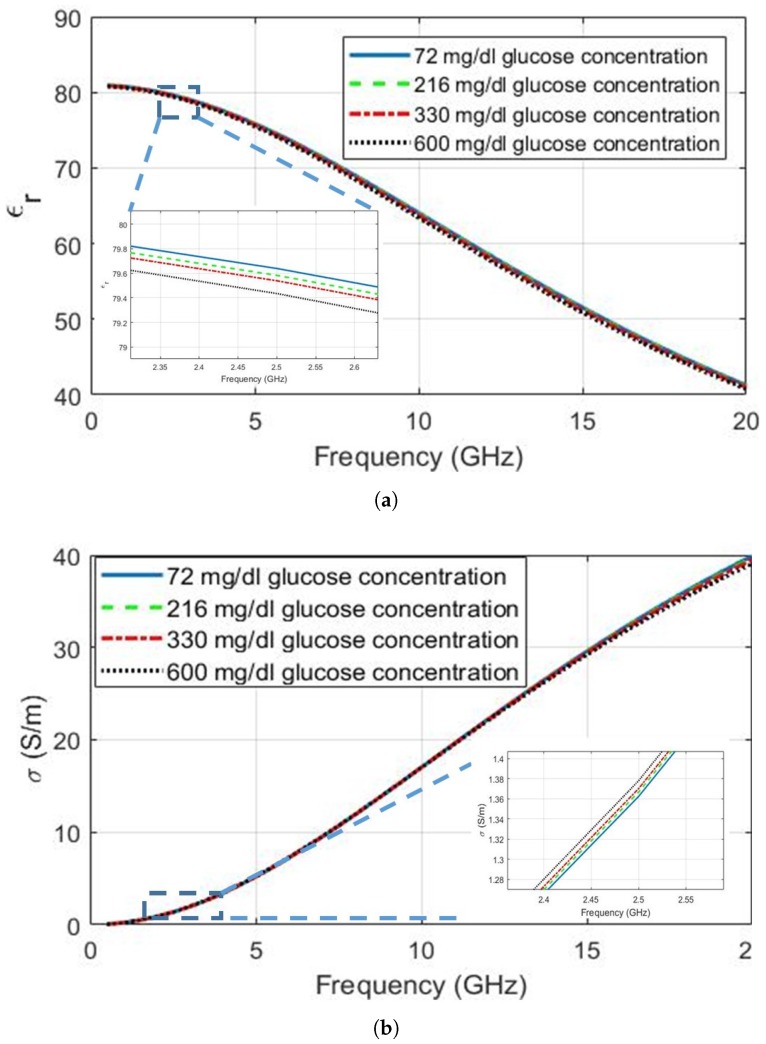
Dielectric properties of water with glucose variations graphed with Debye parameters polynomials given in [33]: (**a**) relative permittivity; (**b**) conductivity.

The approach used in [22] was modified for the Debye parameters and adopted in this study to characterize the glucose-dependent dielectric properties of de-ionized water. Equation (2) and the Debye parameter coefficients are used to obtain Figure 3, depicting the dielectric property change as the glucose levels increase from 72 mg/dL to 600 mg/dL. As seen from the magnified plots inset in the figures, the relative permittivity of the de-ionized water decreases with the increase in glucose concentration, while the conductivity of the deionized water increases. In fact, the observed behaviour of the conductivity changes when the frequency is increased above 9 GHz. The conductivity of the mixture started to decrease with the increase in glucose concentration. Although the trend in relative permittivity agrees with the earlier publications, the trend in conductivity is different when compared to other reported work. As explained earlier, the broadband dielectric property measurement techniques are prone to errors. Since the change in conductivity is expected to be even smaller than the change in permittivity, the conductivity change due to glucose concentration might have been lost.

The dielectric properties at four different frequencies (0.5 GHz, 2.5 GHz, 5.0 GHz and 10.00 GHz) are listed in Table 3, generated using the dielectric property graphs given in Figure 1 and Figure 3. These frequencies were chosen since 500 MHz is close to the Medical Device Radiocommunications Service (MedRadio) and Wireless Medical Telemetry Service (WMTS) bands [36,37], 2.5 GHz and 5.0 GHz are close to license-exempt ISM bands in common use for wearable devices and 10 GHz represents the glucose-dependent dielectric behaviour at higher frequencies. The conductivity of blood plasma is higher than the conductivity of the deionized water at frequencies lower than 10 GHz. This could be due to sodium and other minerals present in the blood plasma. The change in conductivity is very small at all frequencies. Figure 3b does suggest that the conductivity change between 18–20 GHz is greater than other frequencies. It should be noted, however, that measurements at higher frequencies require special equipment and this change might merely due to the greater measurement/fitting errors at higher frequencies. The relative permittivity change is somewhat greater than the conductivity change and seems to be greatest at 10 GHz, among the frequencies shown in Table 3.

This quantitative comparison of models from the literature serves to confirm that the dielectric property changes due to changes in glucose levels are very small. The relative permittivity displays a consistent trend: an increase in glucose level corresponds to a decrease in the relative permittivity of blood plasma and other blood mimicking materials. Since the glucose-dependent relative permittivity change is very small, there is a need to develop structures that are sensitive to dielectric property change and algorithms that can sense the glucose-dependent change among other factors that may affect the dielectric properties of the biological tissue.

In [38], an impedance analyser was utilized in the frequency range of 1 kHz to 1 MHz. Two sets of measurements were performed. The first used aqueous solutions of glucose, with concentrations from 0 mmol/L (0 mg/dL) to 225 mmol/L (4050 mg/dL), at steps of 25 mmol/L (450 mg/dL); hence, this set of measurements did not address the sensitivity issue. It was observed that both the permittivity and conductivity of the glucose solutions were decreasing with the increase in the glucose concentrations [38]. Similar to other studies, the dielectric properties were modelled with the three pole Cole–Cole equation, while the changes in Cole–Cole parameters with respect to change in glucose concentration was modelled with polynomials. The change in the $\Delta \varepsilon_{1}$ plotted to demonstrate that the $\varepsilon_{s}-\varepsilon_{\inf}$ is decreasing with the increase in glucose concentration [38]. The second set of measurements examined the effect of changes in blood volume during the cardiac cycle and used the bio-impedance parameter directly, rather than extracting permittivity and conductivity [38]. These are discussed further in Section 3.2.

### 2.4. Discussion

It is clear, from the qualitative review and quantitative examples given, that the application of dielectric spectroscopy for glucose detection is both possible (as seen by the in vitro experiments) and extremely challenging, due to the small magnitude changes in permittivity associated with the small changes in glucose encountered in realistic scenarios. The next section discusses the issues around selection of the operating frequency and bandwidth.

A few comments must be made here regarding the selectivity issue. All the studies reported on above have examined the behaviour of the dielectric properties under the controlled change of sugar level and our discussion has focussed on the sensitivity issue. In reality, these properties will be affected by other inter-related factors, such as:

- temperature—much of the above research was conducted at standard laboratory temperature and there is little-to-no research available on the combined effect of temperature and glucose-level changes on the dielectric properties. Furthermore, the effect of temperature on various tissues is known to be frequency-dependent with complex behaviour [39];
- perfusion—the volume of blood in the measured region during the measurement period will obviously affect the data and this volume will change with temperature, pulse rate, activity level and clothing (for example, tight sleeves or watch bands can restrict the flow of blood);
- sensor positioning and motion—different locations on the body have been considered for sensor placement (such as the ear lobe, the wrist, the thumb and the torso, as discussed in Section 3.2 and Section 4), either for convenience of testing, comfort for continuous-monitoring scenarios, or for tissue properties at that location (e.g., the ear lobe has a relatively thin skin layer and no bone or muscle). Small motions of the test subject can induce errors in the measurement (e.g., introduction of a small air gap between sensor and skin), potentially even for static test scenarios. For the ideal of continuous monitoring, any sensor must be robust to motion-induced artefacts from small changes in sensor position, as well as related issues (e.g., activity level, contamination of the test site from sweat, dirt and other materials);
- other biological activity—tissues are dynamic inhomogeneous materials, with many bio-chemical and bio-physical process occurring. Examples that may affect the dielectric properties include (but are not limited to) changes in the levels of blood gases (particularly oxygen and carbon dioxide), urea, lactic acid (affected by activity level), as well as changes induced by injury or infection.

Amperometric glucose-sensing, such as used with minimally invasive needle-type bio-sensors, use specific enzymes and filtering membranes to provide selectivity, sensitivity and sensor longevity [2]. A major challenge for non-invasive spectroscopic detection methods is to achieve the selectivity necessary to properly attribute dielectric changes to blood glucose changes.

Another aspect that must be considered is the subject-specific variation in tissues. In particular, there can be significant variation across the population in tissue thickness for various locations on the body, possibly correlating across one or more of gender, age, ethnicity and affluence (and its impact on physical health and fitness), among other factors. Any system that could not accommodate such variation into its model for extracting the dielectric properties would be restricted in application at best. Those systems attempting to penetrate furthest into the body will need to account for variation in muscle and fat tissues, plus other internal tissues (dependent on the location of the device on the body); all systems must account for skin thickness variation. To illustrate this issue, we summarise some key points of a recent paper on measuring the dielectric properties of skin (not aimed at glucose monitoring [12]).

As described in [12] and elsewhere (e.g., [26,40]), there are at least five different ways of modelling skin (ignoring voxelized phantoms from MRI scans). The skin can be considered as a single homogeneous layer in its simplest form, with one effective permittivity. This can be perfectly acceptable and accurate, electromagnetically speaking, depending on the application. Increasing complexity comes by sub-dividing the skin into constituent layers, with the most complex in use being a four-layer model, consisting of the [12]:

- stratum corneum (the outermost and driest layer);
- viable epidermis;
- dermis;
- subcutaneous fat layer.

Intermediate-complexity models come from merging one or more of these layers into homogeneous effective media. (Some also may argue against including the fat layer; from an electromagnetic perspective, there is no particular problem with this, so long as the effective medium averages the constituent media correctly.) The stratum corneum is approximately 20 μm thick, but can be thicker, dependent on body location (and likely varying between subjects to some extent) [12]. The epidermis is normally between 0.06–0.1 mm thick and the dermis is between 1.2–2.8 mm thick [12]. It is noted that a blood layer may be added for glucose-monitoring applications. Furthermore, resonators will typically include at least one dielectric layer in their construction, which must be included in models appropriately.

The models and the effect of the various layers, were investigated numerically between 26.5 GHz and 40 GHz, using models of open-ended coaxial probes and open-ended waveguide (two common probes used in material characterisation) [12]. Further measurements were made at several body locations on three test subjects [12]. The important sources of both modelling and measurement errors were also investigated. Accuracies of up to 85% and 95% were reported for relative permittivity and relative dielectric loss factor, respectively [12].

In addition to this, it has been shown that non-invasive blood glucose sensing at finger-tips is affected by layer thickness and even the presence of small air-gaps caused by finger-prints [41,42]. A shift in resonant frequency of around 20 MHz was observed due to these air-gaps [41] and potentially as much as 100 MHz, depending on fingerprint depth [42]. Given that the air-gaps will differ depending on how the finger is placed on the sensor [41,42], in addition to the effect of pressure on the tissues in the finger tip, it is clear that this effect must be carefully considered, given that frequency shifts as low as 8 MHz [43] could be produced by changes in glucose concentrations, at least for resonators with relatively low Q-factors.

It is also worth noting that the models discussed above assume planar layers of uniform thickness in most instances. The effect of pressure (e.g., of a finger pressed on a sensor, or a smart watch strap on the wrist) will be to reduce the thickness of at least some of the layers in a non-uniform manner, in addition to affecting the flow of blood and interstitial fluid through the tissues.

## 3. Frequency of Choice

### 3.1. An Empirical Approach

Different frequencies have been utilized for blood glucose level detection in the literature, ranging from radio frequencies to millimetre waves. Although some applications at terahertz and infrared range have also been investigated (e.g., [44,45]), the scope of this review is limited to RF/microwave and some millimetre-wave applications. Most of the work reported focusses on narrow-band applications, with a few reporting wide-band behaviour. Since microwave diagnostic and treatment applications are based on the dielectric property discrepancies between the anomalous and healthy tissues, the behaviour of dielectric properties that is dispersive with respect to frequency is one of the primary constraints for frequency of choice.

Another related factor in frequency selection is penetration depth: as conductivity increases with frequency for all tissues, the electromagnetic wave encounters greater loss at higher frequencies, which can be related directly to how much tissue the wave can pass through and still be detectable at the system’s minimum threshold for detection. (A related parameter is the skin depth; we do not discuss this here, but the penetration depth is always greater than the skin depth.) For most implementations of sensors for glucose monitoring, a reflection mode is used; a few use a transmission mode (e.g., for systems placed on the ear lobe). The penetration depth is essentially a reflection-mode parameter; transmission-mode systems can be expected to have a maximum allowed sample thickness approximately twice that of the penetration depth (because the reflected wave passes through the tissues twice). Penetration depth decreases with frequency for all tissues. At low frequencies (e.g., below 100 MHz), the penetration depths for skin, fat and muscle would be more than thicknesses typically encountered; above 10 GHz, however, very little penetration into muscle can be expected, while penetration depths for skin and fat are of less than or equal to typical thicknesses [46]. We note that the limits on exposure, particularly the Specific Absorption Rate [SAR], place an upper bound on transmit power, which translates into a maximum penetration depth for a tissue of given loss.

**Figure 4:**
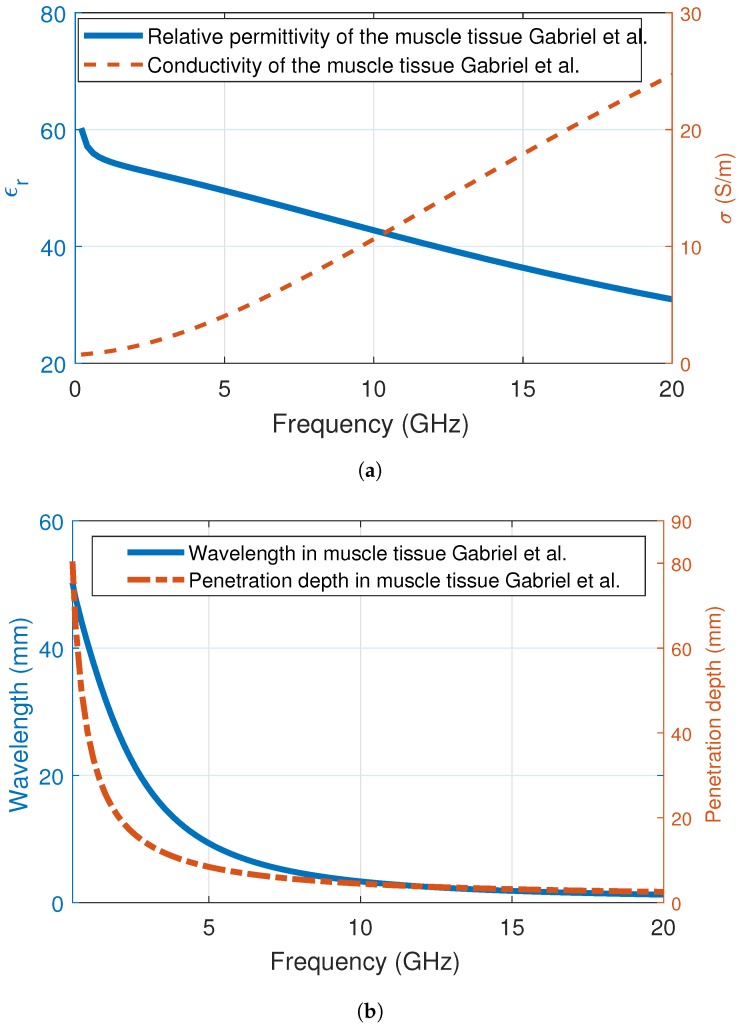
Muscle tissue dielectric properties and wave behaviour in muscle tissue medium: (**a**) relative permittivity and conductivity of muscle tissue between 200 MHz and 20 GHz; (**b**) wavelength and penetration depth in muscle medium.

As noted before, microwave frequencies have been employed for breast cancer imaging and treatment purposes due to the dielectric property discrepancy between the benign and malign tissues [47]. In microwave imaging, the employed frequency range is between 3 GHz and 7 GHz. The resolution of the microwave images increases at higher frequencies; however, penetration depth and wavelength decrease. To illustrate the penetration depth and wavelength in tissue, we plotted the behaviour two values with respect to frequency in muscle tissue. The muscle tissue dielectric properties have been utilized before in the literature to represent the lossy medium of the human body [40]. The relative permittivity and conductivity of muscle tissue is shown in Figure 4a; the change of wavelength and penetration depth in muscle tissue medium with respect to frequency is shown in Figure 4b. Similar to high water-content tissues, the relative permittivity of the muscle tissue decreases with the increase in frequency, while the conductivity of the muscle tissue increases. Both the dielectric property change and the increase in frequency affects the wavelength and penetration depth. From Figure 4b, it can be seen that both the wavelength and the penetration depth is less than 5 mm. This demonstrates that, above 10 GHz, the body tissues will be even more lossy and the propagating wave will quickly attenuate.

One other criterion that can be considered while choosing the frequency of operation is looking into the utilization of the bands. For example, the US Federal Communications Commission’s MedRadio spectrum allocation covers the 413–419 MHz, 426–432 MHz, 438–444 MHz, 451–457 MHz and 2360–2400 MHz ranges. These bands are specifically useful for implants and body-worn devices for off-body, on-body and in-body communications, since the signal is still quite strong for these bands. Other possible bands include the license-exempt 2.4–2.5 GHz and 5.725–5.875 GHz ISM bands. At 2.45 GHz, the wavelength and penetration depth in muscle tissue is around 22 mm for both quantities. At 5.8 GHz, the wavelength and penetration depth in muscle tissue is around 7.6 mm and 7.4 mm, respectively.

Lastly, it can be concluded from Section 2 that the glucose-dependent dielectric property change is very limited, especially in the microwave region, such that the glucose-dependent change does not display a significant variation between the frequencies. This indicates that the limited change in blood dielectric properties due to the glucose variations can only be measured by employing a highly sensitive technique. Broadband dielectric property measurement techniques, such as the open-ended coaxial probe, are known to suffer from accuracy and repeatability limitations, whereas narrow band measurement techniques are known to be more precise. Therefore, empirically we can conclude that a narrow-band technique will be more sensitive to glucose-dependent dielectric property changes. Ultimately, the sensitivity of a resonator also depends on the measurement technique and the performance of the employed technique at the operation frequency. The Q factor, which is indicative of the measurement sensitivity, of narrow-band resonators is expected to increase at higher frequencies (that is, higher order modes for a given resonator tend to have higher Q factors). Considering the constraints imposed by the lossy nature of the biological tissue media (higher loss and smaller penetration depth with increasing frequency) and the band availability, plus the fact that resonators tend to be some multiple of a half-wavelength in size (hence, physically larger at lower frequencies), a narrow-band resonator operating at a narrow band frequency between 4 GHz and 7 GHz can be a viable option. Of course, it is still possible to employ other frequencies, both higher and lower, as can been seen in the literature. We now review these choices, with a discussion following in Section 3.3.

### 3.2. Frequencies Employed in the Literature

Different frequencies have been employed in the literature to sense the glucose change. For example, in [48], a monopole antenna operating between 1–6 GHz was designed. The antenna response between 1.5–3 GHz was observed to shift during simulations and while testing it with blood mimicking phantoms. However, it is known that the response of wideband and ultra-wideband antennas are less sensitive to the dielectric property changes in a medium. For instance, Vivaldi antennas are frequently employed for microwave medical imaging applications to both provide a wideband signal and prevent the antenna detuning due to close proximity to human body. Additionally broadband dielectric property measurement methods known to suffer from low measurement accuracy. Considering that the realistic dielectric property change with respect to change in glucose levels is very limited, there is a need to employ more sensitive techniques.

As mentioned above (Section 2.3), an impedance spectroscopy approach was used in [38]; here, we describe the second set of measurements using the bio-impedance parameter directly, measured using a system from Biopac. Initially, measurements were performed on agar phantoms with aqueous solutions using varying quantities of glucose: 0 mmol/L, 50 mmol/L (900 mg/dL), 100 mmol/L (1800 mg/dL) and 200 mmol/L (3600 mg/dL); again, it must be stated that these are not realistic values, so questions regarding the sensitivity are not addressed. The bio-impedance of glucose solutions with solutions having different glucose concentrations were calculated at 10 Hz and supported the expectation that the change in bio-impedance decreased with increasing glucose concentration. An additional set of measurements were performed on a non-diabetic test subject, in conjunction with direct measurements with a commercial portable blood glucose meter (ACCU-CHEK Performa). Measurements were taken over the course of a 135-minute period, at five-minute intervals for the bio-impedance and thirty-minute intervals for the blood meter, during a type of oral glucose tolerance test. After some signal processing and curve-fitting, it was observed that there was an inverse correlation between the measured bio-impedance and the measured glucose level [38]. This work reported that the temperature, minimum and maximum blood volume and other components of blood (such as haemoglobin) might effect the bio-impedance calculations.

A spiral resonator operating between 628 and 677 MHz was introduced in [30]. This resonator was not tested with realistic glucose values and the response was explored to retrieve the relative permittivity of the tissues. The sensitivity of the resonator can not be judged. It should be noted that the wavelength and penetration depth is quite large at these frequencies. Therefore, the response of the structures operating close to MedRadio bands can be affected by other factors, such as the size of the tissue loaded to the resonator. When the final application is considered, this could emerge as a problem when setting a calibration standard.

Another resonator was presented in [26,43], operating close to 2.45 GHz when loaded with four- and five-layer tissuemimicking materials (composed of dry skin, wet skin, fat, blood and muscle tissue). This resonator, which was not optimised for the wearable glucose monitor application, consisted of a microstrip patch resonator with two capacitively coupled feeding strips and had a Q-factor of around 4, making it relatively poor in terms of sensitivity. The penetration depth and wavelength at this frequency is still quite large (around 20 mm in muscle tissue); therefore, the calibration problem may still emerge at a smaller scale. One option to achieve matching for different loads is to utilize a impedance-matching circuit at these frequencies. However, this approach should be implemented so that glucose-dependent change in impedance will not be masked.

A serpentine-shaped capacitive structure operating between 3.0 GHz and 6.0 GHz was presented in [33,49]. The sensitivity of this structure was analysed for the best matching. Initially, the structure was designed to operate at 4.8 GHz; this frequency was chosen after analysing the penetration depth and reflections between different tissue boundaries. It is worth noting that most of the simulations were performed in commercial electromagnetic simulation programs; when the RF/microwave sensors are loaded with lossy materials (such as four-layered tissue-mimicking materials with frequency-dispersive dielectric properties), the simulations take longer to complete than simulations in air. Therefore, it is advised to run such simulations on workstations; even then, the cost of optimizing these sensors to operate at a certain frequency is high.

Two patch antennas operating at 2.45 GHz and 5.8 GHz were designed and tested with dextrose solutions in an attempt compare the performance of the antennas at these two frequencies [50]. The antennas are mounted at the bottom of two 3D printed cups. The cups were filled with dextrose solutions having concentrations ranging from 0 mg/dL to 5000 mg/dL. From 0 mg/dL to 1000 mg/dL, the amount of dextrose was increased by 200 mg/dL. When the behaviour of the patch antenna was analysed while changing the dextrose amount, it was observed the operation frequency did not change. However, the matching of the antennas changed with the increase in dextrose levels. Although the change was not linear, it was observed that the matching of the antenna operating at higher frequency was more sensitive to the dextrose change. The response of both antennas are given in Table 4. Since the antenna types were identical, the glucose-dependent change in antenna matching is attributed to the frequency of operation.

A resonator operating at 1.4 GHz was proposed in [51]. The sensor, which had a Q-factor of about 800 in air [51], was proposed to be placed on the abdomen region of the body, where its Q-factor was reduced to about 80 [51]. The sensor was tested with humans and the response compared with the commercial glucose sensors. Also, an in vitro interference test technique was proposed to test the sensor performance with glucose and other materials. The resonator response shifted 600 kHz when the glucose levels were increased from 0 mg/dL to 600 mg/dL. The in vitro performance of this resonator does not only depend on the frequency; the structure itself also has an important role. Therefore, in the abdomen region the tissue should not be considered homogeneous in the 1.4 GHz frequency range. Changes in other parameters are likely to affect the resonator response. In [51], the effect of other parameters was mitigated with a reference structure that was separated spatially from the tissue and main resonator, but otherwise identical to it. Hence, the change in resonant response (frequency and bandwidth) for the reference resonator can be used to track temperature via a calibration curve, thus allowing detection of permittivity changes with the main resonator caused by other factors. A clinical trial of this sensor involving 24 human subjects (eight non-diabetics, four Type-1 diabetics and 12 Type-2 diabetics) undergoing an oral tolerance test was reported [52] and assessed using the Clarke Error Grid and the mean absolute relative difference (MARD) parameter. Two versions of the sensor were used (12 subjects per sensor); some differences between sensors were possibly evident, based on MARD values of 11% and 14% for the respective test groups, although no detail is provided on the test subject groups to allow identification of other possible causes. An overall MARD value of 12.5% was calculated. Although the majority of test samples were in regions A and B of the Clarke Error Grid, there were some values in the upper C region (attributed to unexpected movement by the test subjects in [52]), demonstrating further work is necessary to enhance robustness. The comparison in time between the sensor and the reference glucose readings (taken using a YSI 2300 Glucose and Lactate Analyzer) was visually close in both papers [51,52]; curve-fitting models were developed in [51] that have presumably been used in [52] to produce estimated blood glucose levels (in mg/dL) directly.

Another microwave resonator operating at 6.53 GHz was proposed in [53]. When a container of de-ionized water–glucose solution, with a concentration of 0.75 mg/mL, was placed on the resonator, the resonance frequency shifted to 3.43 GHz. The glucose concentration was then increased to 1 mg/mL, then in 1 mg/mL increments to 5 mg/mL. The resonance frequency shifted to 3.53, 3.93, 4.23 and 5.03 GHz for glucose concentrations of 1 mg/mL, 2 mg/mL, 3 mg/mL, 4 mg/mL and 5 mg/mL, respectively. Although a very good resonance shift is observed, it should be noted that the sensitivity can not be merely attributed to frequency of operation. Both the resonator structure and the frequency of operation, thus, the Q factor, are all parameters that needs to be considered in this work. The readers should note the units used, which have a factor of 100 difference to those used in this work, such that the normal range of glucose concentration is stated as from 0.75 mg/mL to 2.16 mg/mL by the authors of [53]. The concentrations used are therefore similar to the values used in this review for quantitative comparisons.

A microstrip-line-based multi-band resonator, operating between 100 MHz and 500 MHz and 1.4 GHz to 1.8 GHz, was proposed in [54]. The resonator was tested using glucose solutions, with concentrations from 0 mg/dL to 5000 mg/dL. The resonance shift, as well as the matching of the resonator, was observed for both frequencies. It was concluded that the resonator displayed a better sensitivity to the glucose change at higher frequencies.

In [55], three versions of a resonator, operating at 1.92 GHz, 5.16 GHz and 7.16 GHz, was designed for measurement of glucose concentrations in microlitre volume solutions. A dielectric sensing cup with a microlitre volume was designed and integrated to these structures to hold the liquid. The Q factor of all three structures was investigated with solutions having different glucose concentrations. The Q factor changed by up to five units for glucose concentration ranging from 0% to 10%.

A spiral resonator was proposed in [56], operating at 7.65 GHz when placed in aqueous glucose solution and operating at 7.77 GHz when placed in a sample of pig blood. During the tests, the sample under test was put into a Petri dish with a diameter of 8 mm, which was placed in turn on the resonator (first, the aqueous solutions were tested, followed by the blood samples). Glucose concentrations for the aqueous solutions were from 0 mg/dL to 600 mg/dL; for the pig blood samples, concentrations from 100 mg/dL to 600 mg/dL were tested. The observed shift in operating frequency was negligible; thus, the authors reported the change in matching of the resonator. For the aqueous samples, with concentrations ranging from 0 mg/dL to 600 mg/dL; the S${}_{11}$ response decreased from −40 dB to −55 dB; for pig blood samples (concentrations ranging from 100 mg/dL to 600 mg/dL), the S${}_{11}$ response decreased from −18 dB to −25 dB. The change in the S${}_{11}$ response with respect to volume was also reported in this work. To the best of the authors’ knowledge, the sample volume should be chosen in such way that the electromagnetic energy completely attenuates within the material under test (MUT) at the operating frequency, in order to explore the true performance of the structure during such experiments. The effective permittivity of the medium measured by the resonator will then only depend on the permittivity of the substrate and the permittivity of the MUT. Since the glucose-dependent dielectric property change is very limited, this is a paramount parameter to explore the full potential of a microwave sensor for blood glucose monitoring.

A metamaterial-based resonator operating at 2 GHz was proposed in [57]. In this work, the change in both amplitude and phase of S${}_{21}$ was tracked. The resonator was simulated with digital phantoms, with the relative permittivity of the digital phantoms changed based on the glucose-dependent dielectric property change equations described in [24]. To simulate the change in blood glucose levels between 0 mg/dL and 250 mg/dL, the relative permittivity of the digital blood-mimicking phantom was changed from 69.4 units to 47.5 units, respectively. When compared to the conclusions drawn in [26], where the change in relative permittivity was expected to be 1 unit for glucose levels from 0 mg/dL to 216 mg/dL, these changes in permittivity values are very large. Since no experimental validation was performed, the performance of the proposed structure can not be fully judged.

The millimetre-wave part of the spectrum, specifically around 60 GHz, is the operating band selected by a company called MediWise for their GlucoWise system [58,59]. This was chosen “*…as the wavelength is short enough for a relatively compact antenna sensor and the penetration depth is large enough for interrogation of thin human tissue regions with sufficient blood concentration*” (Saha et al., 2017 [59]). The developed sensors are intended to work either on the ear lobe or the fleshy part of the hand between thumb and first finger and are based on a pair of patch antennas (resonators) acting in transmission mode. Standard in vitro measurements were conducted using aqueous solutions of glucose and the authors stated the system “*can detect as low as 0.025 wt% of glucose in water*” [59]. Ten non-diabetic male subjects underwent an intravenous glucose tolerance test (IVGTT) while wearing the system. Results for two subjects showed reasonable correlation; poor results for the other test subjects were attributed to hand motion and “*…gradual sliding of the holder during the session, possibly due to fatigue or stress*” [59], emphasising the challenges introduced by external factors. The lag between direct blood measurements and indirect tissue measurements was also evident [59]. A more recent study involving an anaesthetised pig was reported [58], again using an IVGTT to introduce glucose ‘spikes’ to the blood stream. The sensor was compared with a “*spectrophotometric clinical blood chemistry analyzer (iLab 650 by Diamond Diagnostics) and a commercially available glucometer (Contour Next EZ by Bayer)*” [58]. The antennas were located at different positions on the ear of the pig and with varying separations, to investigate variability and the detuning effect. The spikes in glucose level were evident in the sensor response, with a lag of about thirteen minutes. This lag was attributed, in part, to the distance from the injection site and to the known lag between venous blood samples and interstitial fluid. It was also stated that “*although the area is convenient for the sensor placement, it is not particularly rich in blood and contains significant amounts of interstitial fluid*” [58].

### 3.3. Discussion

As seen from the literature reviewed above, there is no settled choice of frequency for non-invasive blood glucose monitoring using dielectric spectroscopy. Some systems use lower frequencies to gain tissue penetration, others use higher frequencies to avoid penetration and most do not specify the reason behind the frequency selection, or else are making it for pragmatic reasons related to design issues, such as device size (a lower frequency means a larger resonator), cost of electronic components (higher frequency components are generally more expensive than lower-frequency components), or wanting to operate in licence-exempt ISM bands. The question of measurement location is affected by and affects, the choice of frequency, via the penetration depth: a finger tip or ear lobe, for example, may require a smaller penetration depth, thus, supporting selection of a higher frequency (e.g., [58,59]). The electronic system must also be capable of sufficient accuracy and precision at the proposed frequency (have sufficient dynamic range and be low-noise).

While there are various arguments for using lower or higher frequencies, it should be mentioned that no system chooses the operating frequency based on the observed spectroscopic behaviour of the glucose molecule. Ideally, measurements would be performed at a frequency ‘near’ to a resonance in the spectroscopic response for glucose, as this would maximise the dielectric property change induced by a change in glucose concentration. We say ‘near’, as it is possible that the human tissues modify the response. Unfortunately, these molecular resonances are almost entirely in the terahertz (THz) part of the spectrum, between roughly 1 THz and the lower end of the infra-red part of the spectrum [10,60]. This is problematic for a number of reasons, including the extremely poor tissue penetration and current technological limitations for operating in this band. Although selective detection of glucose (and other similar molecules) has been successfully demonstrated using THz nano-antennas [60], with resonances between 0.5–2.5 THz, this approach has yet to be translated for work with human tissues, to the authors’ knowledge. Given the poor penetration depth, it remains far from clear that a change in blood glucose concentration could be detected in this band. There would also need to be studies to determine how these resonances appear within tissue samples and whether the resonances of other molecules might obscure that of glucose.

## 4. Utilized Microwave Resonators and Antennas

In this section, we review the resonator geometries utilised in the literature, to try and identify any useful trends. We note that there are different terms used for the microwave sensor in the literature, namely ‘antenna’ and ‘resonator’. Since most antennas are resonant elements, the distinction is somewhat vague (even artificial). Nevertheless, to avoid confusion from the respective camps, we have split the review along these lines, which perhaps arises from the respective backgrounds of the researchers: antennas researchers see the sensor as ‘radiating’ into the body tissues and its response being ‘de-tuned’ by those tissues; resonator designers, perhaps coming from filter design or material characterisation backgrounds, may think in terms of loaded and unloaded Q factors, field distribution across the sample under test and similar things. Essentially, these describe the same responses, but there can be some subtleties in the descriptions.

### 4.1. Antennas

Antennas employed for glucose-dependent dielectric property change include both wide and narrow-band antennas. A wideband monopole antenna operating between 1 GHz and 6 GHz was proposed in [48]. This antenna was simulated with a hand phantom and the S${}_{11}$ response of the antenna was tracked during the simulations. In addition to the drawbacks listed for employing a wideband method, a monopole antenna has an omnidirectional radiation pattern, suggesting that the S${}_{11}$ response of the antenna will be vulnerable to the changes in the vicinity of human body and the antenna. Considering that the glucose levels are only slightly changing the dielectric properties, the variations in S${}_{11}$ response due to glucose-dependent dielectric property change might be lost in uncontrolled environment.

Patch antennas, operating at 2.45 GHz and 5.8 GHz when loaded with deionized water and glucose solutions, are given in [50]. The antennas were printed on FR4 substrate and also covered with an FR4 superstrate to prevent the shorting of the antenna. When the end application is considered, a superstrate can be useful for managing the SAR within the allowed limits. As a side note: since non-invasive measurements are also envisioned for continuous use, the proposed materials should either be built with bio-compatible materials or should be covered with a bio-compatible material. During the experiments, it was observed that the matching of the antennas were changing with the change in glucose levels. The change was non-linear and the antenna operating at a higher frequency was more responsive to the change in glucose levels.

In [61], a patch antenna was proposed, operating at 5 GHz in air and around 2 GHz (depending on glucose concentration) when loaded with a phantom. The antenna was tested with two liquid phantoms, namely physiological solutions and pig blood, with glucose levels ranging from 0 mg/dL to 500 mg/dL. Simulations predicted a linear shift in resonant frequency of 5 MHz for the physiological solution; measurements did not display any correlation, which was attributed to possible differences in temperature and volume between test samples. Simulations using pig blood digital phantom predicted shifts of 200 MHz (comparing 125 mg/dL to 0 mg/dL) and 300 MHz (comparing 250 mg/dL to 125 mg/dL). When the antenna was tested with pig blood, smaller shifts were observed. A linear fit was performed; the resonant frequency increased by 43 MHz when increasing the concentration from 0 mg/dL to 125 mg/dL and from 125 mg/dL to 250 mg/dL and by 86 MHz when increasing glucose concentration from 250 mg/dL to 500 mg/dL. The number of sample points is relatively low, however and further experimental investigations seem advisable.

In [33], a serpentine-shaped antenna with passive coupling was proposed, designed to operate at 4.8 GHz in air. The antenna was envisioned to be placed on the finger tip in the end application. The proposed structure was simulated with a finger model and measured in vivo, with the S${}_{11}$ response tracked (with no monitoring of glucose). It was observed that the antenna had a very narrow bandwidth when operating in air; however, when the antenna was loaded with the finger (finger model), it was observed that the bandwidth increased (a result of the loss of the loading tissues) and it becomes impedance-matched (at the 10 dB return loss level) between 2.8 GHz and 5.5 GHz (or roughly 2.6–3.6 GHz for the simulation). This suggests that the proposed sensor essentially has a wideband behaviour. Since the human body is lossy, the permittivity and conductivity affects the characteristics of the antenna. To quantify the response of the antenna to the change in glucose levels, it was simulated with phantoms representing glucose solutions using de-ionized water. The glucose concentration changed from 0 mg/dL to 2000 mg/dL, resulting in a maximum shift in resonant frequency of 32 MHz. Simulations using a four-layer tissue model of the finger were also performed. The resonance peak shifted from 3.288 GHz to 3.292 GHz when the glucose levels were increased from 0 mg/dL to 2000 mg/dL, a shift of only 4 MHz, demonstrating again the sensitivity challenge.

One other aspect was discussed, namely the effect of the geometry on the electric field, by comparing the proposed serpentine geometry with the patch resonator from [43]. The serpentine resonator was seen to have a higher sensitivity to glucose variation, which is in accord with the idea that geometries with narrow-band responses give more sensitivity. Another way of understanding this is in the effect on the electric field, with the serpentine structure having both greater field intensity and greater field localisation in the central portion of the resonator, when compared with the patch structure. This illustrates another trade-off in the design process: compact geometries tend to achieve greater field intensities in smaller cross-sections, implying greater penetration depths and, potentially, improved sensitivity to changes in effective permittivity. The proviso is that smaller cross-sections also imply less averaging across the monitored volume, implying sensitivity to sensor location (and subject variability) could also increases. (SAR limits obviously still apply, as a constraint on the field intensity.) This study was recently extended in [62] to include the spiral resonator from [30,63] (see Section 4.2), with similar results. The spiral was deemed to be more sensitive than the patch structure and less sensitive than the serpentine structure (again in accord with the Q-factors), with the patch structure achieving greater field intensities. The greater uniformity of electric field for the spiral (compared to the patch) was seen as a contributing factor in the greater sensitivity, as well as in improving measurement uncertainties [62].

### 4.2. Resonators

An open-ended spiral resonator was presented in [63]. The resonator is basically a spiral-shaped microstrip transmission line and has two ports, with two straight microstrip lines capacitively coupled to the spiral line. The spiral shape was chosen to minimize contact orientation errors, due to the symmetry of the structure and to form a standing wave. The amplitude of the standing wave was tracked by measuring the amplitude of S${}_{21}$. The resonator was modified to accommodate typical human thumbs and tested with human subjects. The tests were performed with healthy subjects using an informal oral glucose tolerance test, where the subjects were given a soda drink and the sensor response tracked over time, while the blood glucose levels were tracked with a commercial glucometer. A good agreement was reported in this study.

Following the reported study in [63], an open-ended spiral resonator with direct coupling was designed and tested with flour-and-water phantoms with varying permittivities in [30], as described in Section 2.2. The goal in this study was to retrieve the dielectric properties from the response of the resonator for a single frequency. This is performed by using polynomials that related the S${}_{11}$ response to the permittivity of the material placed on the resonator. The dielectric properties of the material were retrieved around 600 MHz.

A patch resonator operating in the 2.45 GHz ISM Band (when placed on a four-layered tissue-mimicking phantom) was designed and tested in [26]. The input impedance of the resonator was tracked at the operating frequency to quantify the blood glucose change. The blood layer of the four-layered phantom was changed, with the blood-mimicking materials having different concentrations of dextrose. It was concluded that the change in the real part of the input impedance was approximately 0.04% per unit change in glucose concentration of the blood-mimicking material.

Printed resonators operating at three different frequencies were proposed in [55], with dielectric microlitre cups to hold the liquid samples. The resonators were tested with deionized water and glucose solutions, with concentrations ranging from 0% to 10%. The response of the resonator to glucose concentration change was quantified by tracking the Q factor of the resonator. The maximum change in Q-factor (comparing 10% to 0%) was less than seven units, but the response was fairly linear and good agreement between simulation and measurement was observed. This reported study is not suitable for measuring the blood glucose levels continuously, however.

In [51], a ring resonator sensor was proposed. As noted before (Section 3.3), a reference resonator is used to calibrate the sensor for temperature changes in the sensing environment. An interference test system was proposed and the fabricated resonator tested with water solutions to quantify the change in sensor response, both with respect to glucose change and with respect to change of other vitamins and sugars present in the blood. It was concluded that the glucose levels caused greater changes in the resonant frequency and bandwidth of the resonator than other factors, such as ascorbic acid and maltose.

A resonator designed by combining a spiral inductor and interdigital capacitor was introduced in [64]. The resonator was printed on a GaAs substrate and operated at 5.8 GHz in air. The resonator was tested with glucose–water solutions by dropping the solution on the resonator. The experiments were also repeated with the blood plasma (denoted as human serum/human sera) with varying glucose levels. When the blood plasma was placed on the resonator, the resonant frequency shifted to 0.642 GHz; the resonant frequency then increased with the increase in the glucose levels. A sensitivity of 199 MHz per mg/mL change in glucose levels of the sample was reported.

In [53], a cross-coupled stepped-impedance resonator was designed and printed on a GaAs substrate. The resonator operated at 6.53 GHz in air: when loaded with the lossy material (that is, deionized-water and glucose solutions and blood plasma, denoted as human sera in the reported work) with varying glucose levels, the resonance frequency shifted to 3.4 GHz. The sample was dropped on the resonator and the relative permittivity of the samples retrieved from the resonator response. A change in the shunt capacitance of the resonator corresponds to an effective permittivity change and thus, the change in glucose levels. The S${}_{11}$ response of the resonator was tracked; the resonance peak shifted 978.7 MHz per mg/mL change in glucose levels of the sample.

### 4.3. Discussion

The majority of the work reported in the literature has used ‘standard’ geometries (e.g., ring resonators, patch resonators, strip/line resonators, spiral resonators). The geometries with greater Q-factors (more narrow-band responses) show greater sensitivities to glucose changes, as suggested throughout this paper. Resonators with spirals and interdigitated capacitors may have the greater sensitivities [33,62]. There seems to be an opportunity to further investigate the resonator geometries most suited for the glucose-monitoring application. There is also no real discussion of electrode geometries for systems using impedance spectroscopy for glucose monitoring in the engineering literature, although the body of literature for monitoring bio-electric signals (such as from the heart and muscles) may be of use. Some allusion to this issue is also made in patent documents and publications by Biovotion, with minimal or no detail or justification; Biovotion are discussed below in Section 5.1.

One aspect not widely considered or explained in any depth is the effect of resonator geometry on the field distribution around the resonator and into the tissues, the notable exceptions being [33,62]. (Again, some allusion to this issue is also made in patent documents and publications by Biovotion, with minimal or no detail or justification.) This also stands out as a research opportunity.

Although some of the sensors described have undergone some optimisation (e.g., for the intended body location, such as the finger [41,62,63]), much of the reported work has not considered this aspect. This is a gap in the literature, in the opinion of the authors, indicating a research opportunity. This is particularly true for sensors intended for wearable continuous glucose-monitoring systems. Here, there should be some consideration for the form factor of the final device. For wearable systems, there seem to be two main possibilities: a device strapped to the arm (e.g., a smart watch, or a specific device, as in [65,66]) or a ‘smart plaster’ for use on the torso, (upper) arm or (upper) leg. Such a smart plaster could offer greater surface area for accommodating larger resonator structures, but the issue of powering the sensor becomes more complicated.

## 5. Addressing the Selectivity Challenge

Thus far, we have described (in Section 2) the ability to detect changes in glucose level via dielectric spectroscopy and emphasised the challenge of sensitivity. Although there is possibly some scope for improvements in recipes for tissue-mimicking materials, we believe the sensitivity issue has been clearly demonstrated in the literature and that future research must have a greater focus on addressing the sensitivity and selectivity challenges. We further suggest that a multi-band approach seems preferable to single narrow or wide band sensors (Section 3). The justification for this is that narrow band resonators will give greater sensitivity to the observed small changes in effective permittivity than wide-band sensors, but insufficient selectivity against other factors affecting the relative effective permittivity. This limitation would be mitigated, in part, by multiple narrow-band resonances. The research (not to say, commercial) challenge then becomes the selection of the resonant frequencies and realisation of the resonator, which are affected partly by the form-factor and location of the wearable device on the body (Section 4). The use of multiple frequencies is unlikely to be sufficient, however, even with improved electromagnetic models for extracting the permittivity (e.g., compensating for small air-gaps). Additional sensors are almost certainly required to compensate for the ‘external factors’ and substantial (large-scale) studies required to understand the ‘internal factors’ (Section 2.4). In the following, we briefly review some existing approaches to multi-parameter sensing, before discussing how the remaining limitations may be overcome and then describing how this might work within an integrated diabetes management system, through comparison with studies conducted using commercial glucose monitors.

### 5.1. Multi-Parameter Sensing

The monitoring of various physiological signals is of broad interest, with applications in healthcare, sports (elite/professional and amateur), security and space [67,68]. Indeed, the growth of fitness trackers and smart watches attest to the growing trend to monitor personal activity in order to meet personal ‘well-ness’ goals. The value of monitoring such factors for use in glucose monitoring has also been recognised in the literature. For example, Choi et al. account for the effect of temperature via a dual-resonator approach [51,52], while the GlucoWise system “*includes two thermometers (one to measure the sample or skin temperature and one to monitor the ambient air temperature) and a solid-state three-axis accelerometer to detect movement*” (Saha et al., 2017) [59]. This system of additional sensors is not yet used to automatically correct the data, however, which would be required for realistic use.

One of the more developed systems in the literature is the ‘Multi-Sensor’ by Biovotion [65,66,69,70,71,72,73]. This has a long history, with predecessor companies being Pendragon and Solianis (some of the principals for Biovotion were involved in one or both of these predecessors) [10] and patent applications dating back to 2001. The elements of the Multi-Sensor are [65,66]

- dielectric property monitoring using resonators optimised for three frequency ranges:
  - 1–200 kHz, to ‘monitor sudomotor activity’ [65] (that is, sweat monitoring);
  - 0.1–100 MHz, to ‘monitor the effect of glucose variations’ (using three different resonators at the low, high and central parts of the band) [65];
  - 1 GHz and 2 GHz (separate electrodes), to ‘monitor water migration’ [65];
- two temperature sensors;
- one humidity sensor;
- an accelerometer (it is unclear how many axes);
- optical ‘diffuse reflectance’ sensors, to ‘monitor hemodynamic changes’ [65].

**Figure 5:**
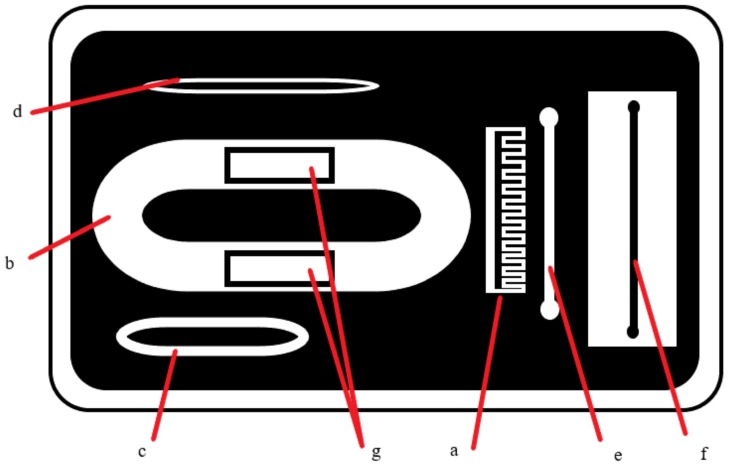
A sketch of the Biovotion Multi-Sensor arm-band, showing the various electromagnetic sensors (some of the sensors described in the text, including the humidity sensor and accelerometer, are within the housing and not visible in this image), based on a drawing from a recent patent document [74]: (**a**) inter-digital electrode operating in 1–20 kHz band as a sweat sensor; (**b**) ‘short’, (**c**) middle and (**d**) ‘long’ MHz-band electrodes; (**e**) ‘short’ and (**f**) ‘long’ GHz electrodes; (**g**) optical reflection sensors consisting of light sources and detectors. (Not to scale; geometries have been simplified) [65,74].

A sketch of the layout of the bottom of a printed circuit board used to form the various resonators is shown in Figure 5, based on drawing of a recent iteration of the Multi-Sensor from a patent document [74]. A number of different geometries have been used, including ring-type, line-type and inter-digitated capacitor ‘electrodes’ (this term is used to describe the Multi-Sensor resonators in the various publications and reflects the impedance spectroscopy perspective).

The Multi-Sensor is designed to be worn on the upper arm and has daily calibration requirements [65,66]. It is relatively bulky and not necessarily suited for continuous monitoring on some practical and aesthetic criteria, but it can be argued that the important task is to develop a fully functioning and reliable non-invasive monitoring device, with such considerations left as future refinements. In the most recent study, twenty Type-1 patients used the system for a total of 1072 study days in home and clinical settings. Training was required to ensure the patients could place the Multi-Sensor on the arm comfortably. One of the objectives of the study was to obtain a larger dataset, over a longer period, than currently available, to guide further refinements and this is an objective that should be considered by other researchers as well.

The results of the study were evaluated using the mean absolute relative deviation (MARD) and the Clarke Error Grid. As expected, there were subject/device-specific variations and a lower accuracy in uncontrolled (home) compared to controlled (clinical) settings. An overall MARD of 35.4 mg/dL was reported, which is still higher than the current state-of-the-art minimally invasive devices (using biosensors); furthermore, although 86.9% of points fell in the A and B zones of the Clarke Error Grid, 0.6% fell in the C zone, 12.1% in the D zone and 0.4% in the E zone. The algorithm used to modify the raw impedance spectroscopy data based on the other sensor inputs is not disclosed in detail, although the improvements in mean absolute deviation (MAD) are discussed. Previous papers suggest the use of principal component analysis and linear regression models (e.g., [73,75]), suggesting one possible source of error is an inadequate sample population. Thus, the potential to detect changes in glucose has again been clearly demonstrated, but not yet in a system that is clinically viable.

### 5.2. Case Study

In this case study illustrating the above issues, we present previously unpublished results from a small-scale study with human subjects conducted by the authors in 2014, investigating the effect of pressure on the response of a patch resonator placed on the wrist. As described above, the patch resonator was previously tested with tissue-mimicking phantoms to verify the proper functioning [26]. The ultimate test domain, to understand the true performance of the structure, requires measurements with human subjects, implying carefully designed experiments to minimize the effects of other changes in the body to the resonator response. One factor that is known to affect the resonator response from earlier observations is the applied force. This effect is due to the change in superstrate geometry and in return effective permittivity changes when the tissue is pressed, squeezed or stretched. Thus, there is a need to calibrate the response of the resonator in order to gather the data relating to the permittivity change due to the glucose levels. However, this approach requires both a multiple sensor system and a smart algorithm to detect the relevant data. As a necessary preliminary step towards this objective, this study conducted initial human experiments performed under controlled conditions. We will use the terms ‘force’ and ‘pressure’ somewhat interchangeably below, possible as the area of the sensor used is fixed.

A measurement platform monitoring both the applied force and the resonator response was built by embedding the patch resonator inside a wooden block and placing two commercial force sensors on the two ends of the resonator. Measurements were performed on one male and four female subjects. The subjects were asked to fast overnight; this was deemed necessary so that the blood glucose levels of the subjects were at the minimum level before the experiment. The effect of the applied force to the resonator response was measured for the different subjects while the blood glucose levels were at a minimum, to establish a baseline and understand the potential measurement uncertainties due to changes in pressure. For this preliminary study, however, the focus was on the development of a robust and reliable test procedure for use in clinical environments; hence, no direct blood glucose measurements were made at this stage.

**Figure 6:**
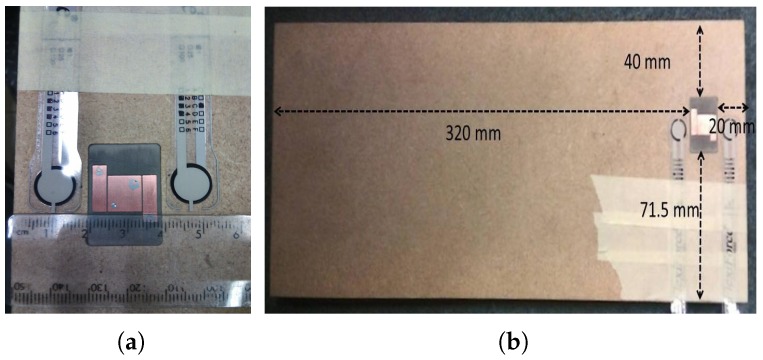
Configuration of the test bench: (**a**) the resonator and force sensors; (**b**) wooden test bench.

The patch resonator presented in [26] was mounted on a wooden test bench with the dimensions of 140.5 × 360 × 18 mm${}^{3}$. The dielectric properties of the wooden bench were measured at the design frequency of 2.45 GHz, giving $\varepsilon_{r}=1.8$ and tan(δ)=0.15. Two force sensors were taped at both end of the resonator, leaving 2 mm space between the resonator and the force sensors. The force sensors were identical and the length, width and thicknesses of the sensors were 100 mm, 14 mm and 0.203 mm, respectively. Both of the force sensors used in this study were A201 type FlexiForce™ commercial thinfilm type sensors [76]. The transparent cover of the commercial sensors was polyester (Mylar) with dielectric properties of $\varepsilon_{r}=3.2$ and tan(δ)=0.005. The experimental configuration of the test bench is shown in Figure 6. The simulation configuration is identical to the experimental set-up. Note that the force sensors were considered as homogeneous Mylar materials during the simulations.

**Figure 7:**
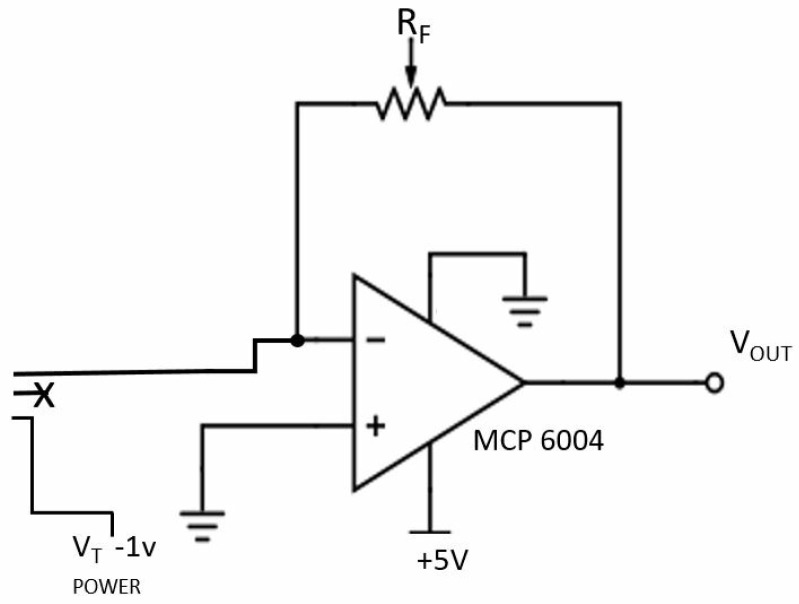
Conditioning circuit for thin-film force sensors [76].

The commercial force sensors can measure applied forces between 0 N and 440 N. The conditioning circuit, used for each force sensor, is shown in Figure 7. The conditioning circuits were built on a breadboard and output voltage of the force sensors were measured with multimeters. The variable resistances were fixed to 203 kΩ.

**Figure 8:**
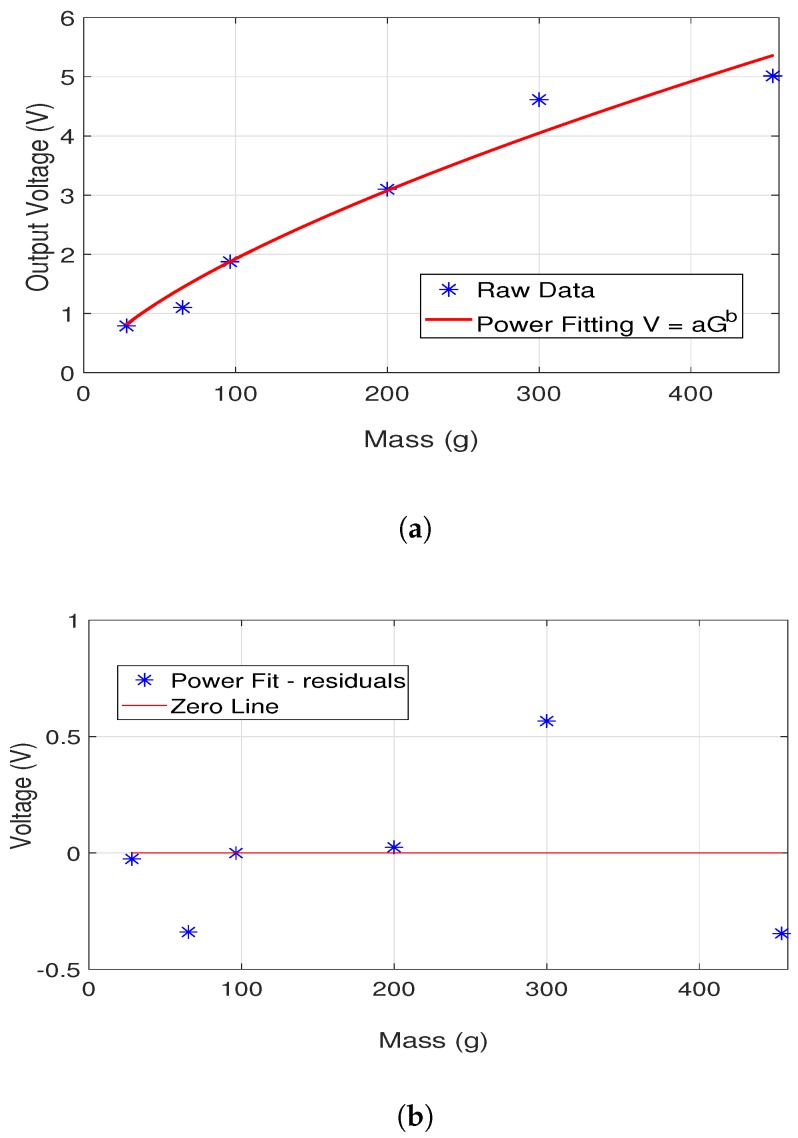
Calibration measurements: (**a**) the power fitting to the calibration values; (**b**) residuals of the power fitting.

The sensors were calibrated to express the applied force, as given by the output voltage, in terms of weight. Calibration was performed by placing a disk of known mass onto the sensing area of the force sensors to concentrate the weight only on the sensing area of the sensors; precise masses were then placed on top of the disk and the output voltages of the sensors measured with multimeters. The calibration graph for the sensor are shown in Figure 8a, where the plotted points are the median values of two sets of data taken from the first and second sensors. The coefficients for the power fitting function is a = 0.085 and b = 0.678. The R-square value is 0.965, quantifying the goodness of the fitting. Residual plots are given in Figure 8b.

Force measurements were performed with one male and four female healthy subjects. The Body Mass Index (BMI) of the each subject is given in Table 5. The age of the subjects were ranging from 25 to 40. The blood glucose levels of the subjects were expected to be constant and low (around 72 mg/dL, or 4–5 mmol/L), as the subjects were fasting overnight before the experiment. The subjects were asked to press the inner part of their right arms to the bench where the resonator and the sensors were mounted. The subjects’ arms were also marked with arm bands, to ensure the same tissue block on each subject was measured and to ensure the placement of the arm matched with the previous measurements for each subject, maximising the repeatability.

**Figure 9:**
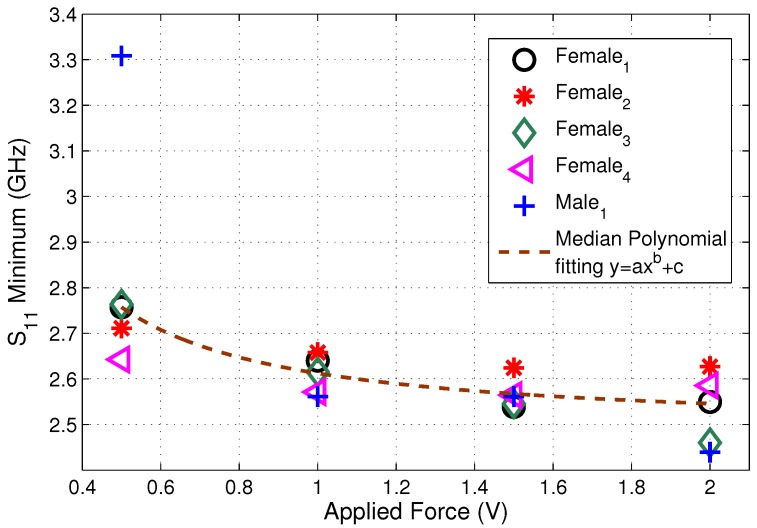
Change in sensor $S_{11}$ response with the applied force voltage output.

During the force experiments, the subjects were asked to apply the same amount of force to both force sensors on either side the resonator. The experimenter recorded the resonator response when the same level of force was reached on both sensors (implying an equivalent force was applied evenly across the resonator). For each force level, the resonator response was recorded five times. The subjects applied four different levels of force as determined by the force sensor output voltages, from 0.5 V to 2 V in 0.5 V increments, with force determined from the calibration curve in Figure 8a when required (the results given below are expressed in terms of the directly measured sensor output voltages). The median of the collected response was taken for each force level for each subject, shown in Figure 9. In Figure 9, the red curve, expressed by $y=ax^{b}+c$ where a=0.12, b=−1.13, c=2.49, shows the median of all measurements.

The median fitting indicates that the superstrate permittivity increases with the increase in applied force. When the applied force is very low, for example at 0.5 V output, an air gap might be introduced between the tissue and the resonator, minimising the effect of the tissue superstrate on the resonator output (note that the resonance shift has a 5.3% decrease between 0.5 V and 1 V outputs). As the applied force increased, the magnitude of the resulting resonance shift decreases. The decrease in resonance is 1.7% and 0.8% for an increase in applied force from 1 V to 1.5 V and from 1.5 V to 2 V, respectively. This could be due to the tissue displacement: it is hypothesized that the fat tissue is displaced with the increase in applied force (also suggested recently in the literature [42,62]). This experiment was important to assess the effect of the applied force to the resonator. From the change observed in Figure 9, it is clear that the applied force has a significant effect on the resonator response; thus, it should be kept constant to differentiate the effect of the glucose change to the resonator response, ideally. In real-world scenarios, appropriate models should be utilised to remove the effect of pressure changes. Note that during the experiments subjects were not be able to apply greater forces consistently (the sensor output can reach up to 5 V).

After the force measurements were completed, the subjects were asked which force level was most comfortable. The subjects reported that the 1 V output was the most comfortable force level. The standard deviation from the mean (σ) on each applied force level among the five measurement was also calculated and are shown in Table 6; the 1 V applied force level shows least deviation. Although obviously only a small and unrepresentative sample, this type of information could be useful in the design stage for resonators and effective permittivity models, by looking for deviations from the target pressure and having models for changes in tissue properties (such as from compression) and air gaps.

### 5.3. Discussion

We have reached a point in this review where we believe we have demonstrated that the detection of glucose via dielectric spectroscopy requires highly sensitive systems that can account for a large number of external factors (such as temperature variability, the effect of pressure and the effect of sweat) to accurately determine the effective permittivity of glucose. This is independent of the frequency (or frequencies) used for the spectroscopic measurement, the optimum choice of which is far from clear. The fact that systems accounting for one or more of these external factors (e.g., [51,52,65,66]) still fall short of the required accuracy for acceptance by clinicians and regulatory bodies demonstrate that it is the biological factors—what we have called ‘internal factors’, plus issues of variation between subjects—that are the main hurdle (although we do not rule out the possibility for improvements in compensation of external factors, we do not believe these to be the primary limitation at this stage). Unfortunately, we do not believe that some clever design of wearable resonator operating at some ‘special’ frequency will overcome these limitations; hence, microwave engineers and other electromagnetic experts will not be able to address this challenge alone. We reiterate we are considering wearable microwave sensors for non-invasive monitoring; we are not considering implants, or whether THz sensors near the molecular resonance of glucose may be able to operate on the skin successfully. Even for these cases, of course, we would expect strong, multi-disciplinary teams to be necessary; it is possible, however, that the problems of sensitivity and selectivity would be less severe in these scenarios.

One possible research strategy to find answers to the above issues is as follows: in addition to continued improvements in capturing good permittivity data and isolating ‘unexplained’ variations from variations explained by other sensor outputs, more work is required to look at how the tissue permittivity varies with changes in other substances in tissues, both in vitro (including the use of phantoms) and in vivo. This will help build up a mass of knowledge that can be used to design better regression models, or even use in machine-learning and artificial intelligence techniques (e.g., fuzzy logic classifiers, neural networks and decision trees [8]), to build predictive systems. Real-time tracking is obviously important, but it is predictive capability that will save lives by warning of potential hypo- and hyperglycaemic events [10]—even if the action by (initial versions of) the system is to warn the user to confirm blood glucose level via a blood sample. Recent improvements in the use of technology in medical studies—such as the use of Apple’s HealthKit, CareKit and ResearchKit (e.g., [77,78]) to capture data, the improvements in ambulatory monitoring devices in comfort and form-factor, the use of commercial wearables to provide activity and other data (although the accuracy of such systems has been questioned, they appear adequate, with the possible exception of low-intensity activities [79,80,81])—should all be leveraged to help achieve the required dataset. Commercially-available minimally invasive systems from the likes of Abbott, Dexcom and Medtronic now communicate with smart phones and to the cloud, allowing diabetics (as well others, such as spouses and healthcare professionals) to receive alerts for high and low glucose excursions, improving patient outcomes and peace-of-mind. A similar approach, potentially capturing other data, could be used to collect the necessary dataset across a wider sample population (e.g., [82,83,84]). Personalised models have been shown to have greater predictive power than global models (e.g., [73]), implying a period of calibration or learning by the system to realise the full potential. This would be in conjunction with traditional monitoring by blood samples and transition the system from the (acceptable but less accurate) global model to the personalised model.

## 6. Conclusions

In this review article, we have sought to convey both the potential and the challenges associated with measuring blood glucose levels non-invasively via microwave dielectric spectroscopy. We have shown (qualitatively, via the review of the literature and quantitatively, by applying realistic glucose levels to models from the literature) how changes in glucose levels produce only small changes in the dielectric properties, even under controlled conditions. This implies a highly sensitive sensor will be required to detect the small changes, which in turn implies low-noise electronics and a ‘high-Q’ (narrow-band) resonator to reduce the impact of noise and maintain the system dynamic range.

We have further discussed the issue of selectivity: the measured dielectric properties are the ‘effective’ properties of a medium equivalent to an average of the constituent tissues, each of which has biological process that affect the individual contributions. These ‘internal factors’ are temperature- and frequency-dependent and are coupled with ‘external factors’ that include such things as external temperature, activity level and even the fit of the clothes worn. A accurate method for retrieving the effective permittivity of the tissue(s) from the resonator response was briefly discussed (Section 2), but this is independent from relating the effective permittivity to the blood glucose level. We have described a number of ‘multi-parameter’ sensing strategies designed to mitigate or remove the effect of the ‘external factors’ and discussed the challenges of dealing with the ‘internal factors’.

Given these challenges, it may seem that dielectric spectroscopy is unsuitable for the task at hand. Indeed, with the decision to suspend activities into a smart contact lens for glucose monitoring by Verily (part of Alphabet, owner of Google) [85], the dream of non-invasive monitoring may seem unachievable (although the early work by Noviosense, which also works with tears and notably uses an enzyme-based approach to achieve selectivity, offers some hope [9,86]). Rather than concede this, we have suggested avenues for further exploration that may enable true non-invasive, continuous monitoring of blood glucose levels to be achieved. In particular, we have suggested that a number of frequencies must be monitored and combined with data from other sensors in a suitable fashion.

Admittedly, there may be some scepticism [10] that adding additional sensors will overcome the fundamental issues of the low sensitivity of the effective dielectric to changes in blood glucose level and the sensitivity of the dielectric properties to other factors and this cannot be dismissed out-of-hand. On the other hand, it is certainly true that signal processing techniques can be used to ‘clean up’ a received message or radar return in order to extract low-amplitude variations in the presence of large-magnitude variations, given suitable measurements and models. Furthermore, there are now methods for identifying otherwise unrecognisable patterns in data that could help tease out the relationship between blood glucose level and effective permittivity, given a large enough dataset. As the availability of computational resources and machine-learning techniques is far greater now than even five years ago, as are new ways of collecting the data required from a suitably large sample population at a reasonable cost (e.g., using Apple’s ResearchKit, coupled with smart watches that can collect other data, including heart rate and activity level). We suggest that there is still scope for progress, but believe that it will ultimately require even greater cross-disciplinary collaboration than seen to date. It has been suggested [10] that, for non-invasive continuous monitoring of blood glucose to be realised,

“…a reasonable chance at success requires in-depth knowledge of all the following disciplines:

- The engineering disciplines related to [the] primary technology, e.g., optics, electronics, software, mechanical engineering, etc.
- Biochemistry, especially knowledge of the glucose molecule and its relation to the chosen field of technology.
- Physiology, especially the distribution of glucose in fluids and tissue.
- Metabolism, especially glucose sources and sinks.
- Diabetes, especially aspects of the disease that will affect [the primary] technology—the more understanding of endocrinology, the better.
- The history of non-invasive investigations, especially in [the primary] technology field—what didn’t work and why.
- The regulatory requirements for a diagnostic device and the evolving structure of the market for existing devices.”

(Smith, 2018 [10])

We also suggest the engineering expertise required must include specialists in signal processing and computational intelligence, as well as microwave and electromagnetics specialists, medical professionals and patients. Attention should focus on the design of compact, highly sensitive resonators (preferably at multiple frequencies, although the selection of these frequencies remains an open research question), coupled with methods of removing the effect of the various external factors from the measured signal and then analysing the signal in the context of a sufficiently large dataset to extract reliable glucose data while accounting for subject-specific variations and the complex internal factors also affecting the effective permittivity.

## Figures and Tables

**Table 1:** Recipes of Tissue-Mimicking Materials including low, intermediate and high water-content tissues from 300 MHz to 20 GHz [26] (food colouring is used to distinguish the materials).

Ingredient (g)	Wet Skin	Fat	Blood	Muscle
Deionized Water	230.0	57.4	230.0	230.0
Gelatine	34.1	15.0	34.1	34.1
NaCl	1.4	0.0	1.2	1.2
Oil	75.0	329.6	15.0	35.0
detergent${}_{1}$	40.0	0.0	40.0	40.0
detergent${}_{2}$	0.0	10.0	0.0	0.0
food colouring	1.3	0.0	0.0	1.3

**Table 2:** Polynomial coefficients fitted to the Debye parameters [33]. Reproduced with permission from Turgul V., Kale I., Sensors and Actuators A: Physical, Elsevier, 2018.

*u*	$a_{n}$	$b_{n}$	$c_{n}$
$\varepsilon_{\infty }$	−8.214 × 10${}^{-8}$	2.148 × 10${}^{-3}$	8.722
$\varepsilon_{s}$	2.318 × 10${}^{-9}$	−2.793 × 10${}^{-4}$	81.015
τ	−8.370 × 10${}^{-9}$	5.150 × 10${}^{-4}$	8.776

**Table 3:** Dielectric property change of blood plasma (BP) and water–glucose with respect to glucose concentration at 0.5, 2.5, 5.0 and 10 GHz based on the Cole–Cole and Debye parameter polynomials given in [22,33]. (*d-water*—de-ionised water)

Glucose	Frequency	$\varepsilon_{r}$	$\varepsilon_{r}$	σ	σ
Concentration	(GHz)	BP	*d-water*	BP [22]	*d-water* [33]
(mg/dL)				(S/m)	(S/m)
72	0.50	72.75	80.94	2.065	5.55 × 10${}^{-2}$
219		72.73	80.9	2.046	5.57 × 10${}^{-2}$
330		72.71	80.87	2.030	5.58 × 10${}^{-2}$
600		72.66	80.79	1.995	5.62 × 10${}^{-2}$
72	2.50	69.74	79.64	3.498	13.62 × 10${}^{-1}$
219		69.70	79.58	3.482	13.67 × 10${}^{-1}$
330		69.67	79.54	3.470	13.70 × 10${}^{-1}$
600		69.59	79.43	3.441	13.78 × 10${}^{-1}$
72	5.00	64.62	75.86	7.078	51.59 × 10${}^{-1}$
219		64.56	75.76	7.069	51.71 × 10${}^{-1}$
330		64.51	75.69	7.062	51.80 × 10${}^{-1}$
600		64.39	75.51	7.046	52.01 × 10${}^{-1}$
72	10.00	53.24	64.07	16.91	17.00
219		53.14	63.90	16.91	17.00
330		53.07	63.76	16.91	16.99
600		52.88	63.44	16.90	16.97

**Table 4:** Comparison of patch antennas operating at 2.45 GHz and 5.8 GHz tested with deionized water–dextrose solutions [50].

Dextrose Levels (mg/dL)	S${}_{11}$ Response (dB) at 2.45 GHz	S${}_{11}$ Response (dB) at 5.8 GHz
0	−14.87	−26.32
200	−14.82	−30.19
400	−15.4	−38.85
600	−14.45	−38.52

**Table 5:** Body mass index (BMI) of the subjects.

Subject	BMI	Subject	BMI
$Female_{1}$	22.1	$Female_{4}$	21.9
$Female_{2}$	25.0	$Male_{1}$	22.1
$Female_{3}$	22.5		

**Table 6:** Standard deviation from the mean (σ) on each applied force level.

Force	Female${}_{1}$	Female${}_{2}$	Female${}_{3}$	Female${}_{4}$	Male${}_{1}$
(V)	σ (MHz)	σ (MHz)	σ (MHz)	σ (MHz)	σ (MHz)
0.5	25.954	9.5513	24.954	13.257	29.681
1	4.2943	4.6416	8.5015	1.1808	12.343
1.5	17.162	4.7459	25.373	12.159	10.428
2	6.7883	2.4348	2.7803	0.88977	19.459
